# Instruments to evaluate non-technical skills during high fidelity simulation: A systematic review

**DOI:** 10.3389/fmed.2022.986296

**Published:** 2022-11-03

**Authors:** Orsola Gawronski, Kiara R. Thekkan, Catia Genna, Sabrina Egman, Vincenza Sansone, Ilaria Erba, Alessandro Vittori, Carmelita Varano, Immacolata Dall’Oglio, Emanuela Tiozzo, Fabrizio Chiusolo

**Affiliations:** ^1^Professional Development, Continuing Education and Research Unit, Bambino Gesù Children’s Hospital (IRCCS), Rome, Italy; ^2^Clinical Risk, Innovation and Integration of Care Services, Bambino Gesù Children’s Hospital (IRCCS), Rome, Italy; ^3^Department of Anesthesia and Critical Care, Bambino Gesù Children’s Hospital (IRCCS), Rome, Italy; ^4^Department of Pediatric Cardiology and Cardiac Surgery, Bambino Gesù Children’s Hospital (IRCCS), Rome, Italy

**Keywords:** high fidelity simulation, non-technical skills, crew resource management, teamwork, human error, psychometrics, assessment and evaluation, reproducibility of results

## Abstract

**Introduction:**

High Fidelity Simulations (HFS) are increasingly used to develop Non-Technical Skills (NTS) in healthcare providers, medical and nursing students. Instruments to measure NTS are needed to evaluate the healthcare providers’ (HCPs) performance during HFS. The aim of this systematic review is to describe the domains, items, characteristics and psychometric properties of instruments devised to evaluate the NTS of HCPs during HFS.

**Methods:**

A systematic review of the literature was performed according to the Preferred Reporting Items for Systematic reviews and Meta-Analyses (PRISMA). Studies were retrieved from PubMed, Cinahl, Web of Science, Cochrane Library, ProQuest and PubPsych. Studies evaluating the measurement properties of instruments used to assess NTS during HFS training were included. Pairs of independent reviewers determined the eligibility, extracted and evaluated the data. Risk of bias and appraisal of the methodological quality of the studies was assessed using the Consensus-based Standards for the selection of health Measurement Instruments (COSMIN) checklist, and the quality of the evidence with the Grading of Recommendations, Assessment, Development and Evaluation (GRADE).

**Results:**

A total of 3,953 articles were screened. A total of 110 reports were assessed for eligibility and 26 studies were included. Studies were conducted in Europe/United Kingdom (*n* = 13; 50%), North America/Australia (*n* = 12; 46%) and Thailand (*n* = 1; 4%). The NTS instruments reported in this review included from 1 to 14 domains (median of 4, Q_1_ = 3.75, Q_3_ = 5) and from 3 to 63 items (median of 15, Q_1_ = 10, Q_3_ = 19.75). Out of 19 NTS assessment instruments for HFS, the Team Emergency Assessment Measure (TEAM) can be recommended for use to assess NTS. All the other instruments require further research to assess their quality in order to be recommended for use during HFS training. Eight NTS instruments had a positive overall rating of their content validity with at least a moderate quality of evidence.

**Conclusion:**

Among a large variety of published instruments, TEAM can be recommended for use to assess NTS during HFS. Evidence is still limited on essential aspects of validity and reliability of all the other NTS instruments included in this review. Further research is warranted to establish their performance in order to be reliably used for HFS.

## Introduction

Adverse events and deaths due to human error are still significant in different fields of healthcare in spite of diagnostic advancements and their therapeutic options (1). Errors during emergency situations on hospital wards have been found to be related not to medical knowledge but to the way this is applied in complex and multidisciplinary settings (2, 3). Various reports point out that human factors contribute to 43–70% of adverse events in emergency and operating room settings (4–9). Communication breakdowns, lack of leadership and teamwork, lack of knowledge of the work environment and failed closed loop communication affect the patient care process in acute and intensive care wards (10, 11).

“Crisis resource management” (CRM) is a simulation-based training program adapted from aviation to healthcare teams for teaching non-technical skills (NTS) to healthcare providers (HCPs) and optimize team performance during patient emergencies and critical events (12–14). NTS are interpersonal cognitive, social and personal management skills that are an adjunct to technical skills (TS), contributing to safe and efficient task performance (15–17). NTS involve effective teamwork, leadership, communication, decision making, situational awareness, task and resource management (12, 18–21).

Simulation training of resuscitation team members is highly recommended by the American Heart Association (AHA) and International Liaison Committee on Resuscitation (ILCOR) (22, 23). High fidelity simulation (HFS) training enables the acquisition of critical thinking, TS and NTS through experiential learning using sophisticated life-like manikins in a realistic patient environment. High fidelity simulations are characterized by a high level of realism associated with the simulation activity, including physical (environment, equipment), psychological (emotions, situational awareness) and social factors (group culture, goals and motivations) (24, 25). Simulation-based training in pediatric critical care settings has resulted in improvements in knowledge and safety attitudes by reflecting on clinical situations and early recognition or management of conditions of risk to patient safety (26–29). Simulators and audio/video-recording of simulation training enable participants and teams to improve their skills through debriefing and replay. NTS are increasingly evaluated in medical and nursing students to evaluate their competences and the effect of simulation training on the acquisition of NTS (28).

The identification of valid and reliable instruments for the evaluation of NTS provides an opportunity to standardize their evaluation in HFS programs and avoid measurement errors (30). To date, several tools have been developed for this purpose but no gold standard has been established to evaluate NTS. The aim of this review is to identify and describe the domains, items, characteristics and psychometric properties of published instruments to evaluate NTS of HCPs during HFS.

## Methods

### Study design

The systematic review was conducted according to the Preferred Reporting Items for Systematic reviews and Meta-Analyses (PRISMA) (31). Our review aims to answer the following research questions: “What are the characteristics of published instruments to measure team NTS during HFS in healthcare?”; “What are the measuring properties of those instruments?”; “Are the instruments valid and reliable?”

### Search strategy

A systematic search was performed on the following databases: PubMed, US National Library of Medicine, by National Center for Biotechnology Information (NCBI), CINAHL Cumulative Index to Nursing and Allied Health Literature, by EBSCOhost, Web of Science Core Collection™ by Clarivate, Cochrane Library by The Cochrane Collaboration, ProQuest by ProQuest LLC and PubPsych by applying the filter “Human” to the search, which we conducted in July 2021 with no time limits and updated in September 2022.

The key words identified and used to formulate the search strategy were: “Simulation Training,” “High Fidelity Simulation Training,” “Assessment,” “Evaluation,” “human factor*,” “resource management,” “stress management,” “resource utilization,” “task management,” “human error,” “non-technical skill*,” “nontechnical skill*,” “Intersectoral Collaboration,” “Crew Resource Management, Healthcare,” “Leadership,” “Decision Making,” “Situation awareness,” “Communication,” “Team work,” “Team-work,” and “Teamwork.” The PubMed search strategy was peer-reviewed by a PhD prepared nurse, expert in systematic reviews. The search strategy for PubMed, CINAHL, and Cochrane is reported on Supplementary Table 1.

### Eligibility criteria

Studies eligible for inclusion reported the characteristics, validity or reliability of NTS evaluation instruments applied to HFS training in healthcare. NTS included communication skills, leadership, teamwork, situation awareness, decision making and task management.

The inclusion criteria were the following: (1) articles describing the characteristics, the measurement properties and performance of NTS evaluation instruments applied to HFS in healthcare; (2) instruments designed for use by direct observations of or audio-visual recordings of HFS.

The exclusion criteria were: (1) different context from healthcare training; (2) low fidelity simulations defined as: simulations using role playing or task trainers designed for specific tasks or procedures for student learning “not needing to be controlled or programmed externally for the learner to participate” (25, 32); (3) no evaluation of the instruments’ validity or reliability; (4) evaluation of TS only; (5) systematic reviews; (6) unavailability of full texts; (7) language other than English.

### Study screening

**FIGURE 1:**
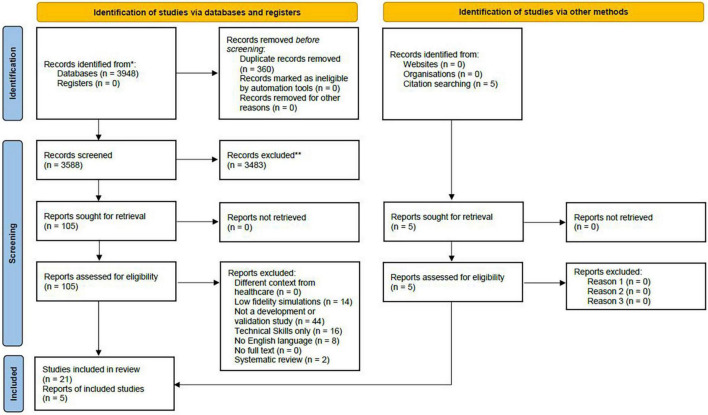
PRISMA Flow diagram of article screening and selection process.

Six independent reviewers screened the titles and abstracts for eligibility according to inclusion criteria, followed by the full-texts of articles identified as “included” or “interesting.” Each article was evaluated on a double-blinded basis by two researchers independently. The Rayyan Intelligent Systematic Review System was used to perform blinded electronic screening (33). Preliminary training on its use was performed on a selected database. Disagreements at each level of screening were resolved through consensus discussion or assistance by another reviewer if needed. Duplicate records were identified and removed. The search process and number of articles retrieved and excluded at each step of the process are shown in the PRISMA flow diagram (Figure 1).

### Data extraction

Data were extracted from each paper included in the review by two authors independently using three tables on Microsoft Word. Extracted information was verified by a third reviewer. Data extracted included: n° of domains, items and type of NTS, scale used to describe the characteristics of NTS instruments; country, study objective, population, type of scenario, the psychometric properties assessed according to the COSMIN criteria, and the final GRADE assessment.

### Quality assessment

#### Methodological quality of included studies by measurement properties

The methodological quality of the included articles was evaluated using the Consensus-based Standards for the selection of health Measurement Instruments (COSMIN) checklist (34–36).

The COSMIN checklist is a validated and standardized quality assessment tool and is increasingly used in systematic reviews of instrument measurement properties. The COSMIN checklist provides clear evaluation criteria and standards for the methodological quality of studies that report and evaluate the psychometric properties of measurement tools. Over a total of 88 items, 70% are related to the study design phase (35). The COSMIN method requires assessing the methodological quality of each study across 10 boxes, including questions referred to tool development, content validity, structural validity, internal consistency, cross-cultural validity, reliability, measurement error, criterion validity, hypothesis testing for construct validity and responsiveness.

The research team met to review and discuss the COSMIN criteria, methods and ratings to ensure a standardized approach in accordance with the guidelines. Six research team members independently scored, in sub-teams of two, each set of questions on a 4-point rating scale (“Inadequate,” “Doubtful,” “Adequate” or “Very Good”) based on the COSMIN criteria and checklists. Standardized forms in Excel were used to report the assessments. Any disagreement in the ratings were resolved by negotiation by the two reviewers, with a third reviewer involved when necessary. In accordance with the COSMIN guidelines, methodological quality scores for each NTS instrument were assigned by taking the lowest rating of any item in each box.

#### Overall rating of non-technical skills instruments by measurement properties

To obtain an overall rating on the identified NTS instruments, the results obtained for each measurement property were evaluated. Based on the criteria for a good measurement property, each result obtained from each study was assessed as “sufficient,” “insufficient,” “inconsistent,” or “indeterminate.” Consistent results of more studies on the same NTS instrument were grouped together and the overall rating (OR) on measurement properties of each identified NTS instrument was determined, according to the COSMIN criteria.

#### Quality of evidence of non-technical skills instruments by measurement properties

Finally, we used a modified GRADE (Grading of Recommendations, Assessment, Development, and Evaluation) approach to ensure strength and certainty of evidence. The quality of evidence for each measurement property was graded as “high,” “moderate,” “low” or “very low” based on methodological quality and overall rating, according to the COSMIN manual.

## Results

### Study selection

Our search produced a total of 3,953 potentially eligible studies. After duplicates were removed, the titles and abstracts of the remaining 3,593 studies were screened. A total of 110 papers were assessed for eligibility, and 26 were included in the review as relevant to the research question (Figure 1).

### Study characteristics

In this review, 19 NTS assessment instruments for HFS were found. Studies were conducted in the following regions or countries: Europe/United Kingdom (*n* = 13; 50%), North America/Australia (*n* = 12; 46%) and Thailand (*n* = 1; 4%).

All studies involved simulation training with HCPs or students that practice in hospital and/or university settings. The sample size of the included studies was between 5 (37) and 177 HCPs/students (13). Four studies did not report the number of participants involved (38–41). HCPs included physicians (4 studies; 16%), midwives (1 study; 4%), medical or nursing students (9 studies; 34%), and multiprofessional teams of HCPs (12 studies; 46%). The NTS instruments were used during simulations of in-hospital patient emergencies. Simulation scenarios focused on general (*n* = 9; 36%), surgical (*n* = 4; 16%), obstetric and gynecological (*n* = 4; 16%), pediatric (*n* = 3; 12%), trauma (*n* = 3; 12%) and operating room emergencies (*n* = 2; 8%). One study did not report the type of simulation scenario. Supplementary Table 2 reports the NTS instruments ordered by type of scenario.

The NTS instruments reported in this review included 1 to 14 domains (median of 4) and 3–63 items (median of 15). The most frequent domains were Leadership (16; 84%), Teamwork (14; 79%), Situational awareness (13; 68%), Communication (13; 63%), Task management (9; 48%), and Decision making (9; 47%). All the instruments used a Likert scale to measure the assessment, with scores that ranged from a minimum of 0 to a maximum of 9 points (median of 1–5 Likert point scale). The characteristics of the included studies and NTS instruments are reported in Tables 1, 2.

**TABLE 1:** Characteristics of the included non-technical skills instruments.

NTS instruments	N° of domains	N° of items	Teamwork	Leadership	Communication	Decision making	Situation awareness	Task management	Other variables	Likert scales (range)
**ANTS**	4	15	✓			✓	✓	✓		1–4
**ANTSdk**	4	16	✓	✓		✓	✓			1–5
**ASNTS**	3	3	✓	✓				✓	Problem solving; Team orientation	1–5
**Assessment of EM physicians’ NTS**	4	12	✓	✓		✓	✓			1–9
**AOTP-GAOTP**	6	18	✓		✓		✓	✓	Environment in the room Communication with patient and partner	1–5
**BARS tool**	4	4	✓		✓	✓	✓			1–9
**CALM**	4	16	✓	✓	✓			✓		1–4
**Ottawa GRS**	5	6		✓	✓		✓	✓	Problem solving	1–7
**HFRS**	5	45	✓	✓	✓				Confidence assertion; error	1–5
**LOSA**	4	13		✓	✓		✓		Preoperative preparation	1–5
**MHPTS**	1	16	✓	✓	✓					0–2
**NOTSS**	4	12	✓	✓		✓	✓			1–4
**NTS-NAS**	14	63	✓	✓	✓	✓	✓	✓	Know the environment, Call for help, Distribute the workload, Use all information, Prevent fixation errors, Cross check, Use cognitive aids, Allocate attention	1–5
**OSANTS**	7	7	✓	✓	✓	✓	✓		Professionalism, Managing and Coordinating	1–5
**OSCAR**	6	48		✓	✓	✓	✓		Cooperation and Coordination	0–6
**STAT**	3	26	✓	✓				✓	68 Technical Skills items	0–2
**T-NOTECHS**	1	5		✓	✓	✓	✓	✓		1–5
**TEAM**	3	11	✓	✓				✓		0–4
**TPOT**	5	25	✓	✓	✓		✓		Team Structure	1–5

ANTS, Anesthetists’ Non-Technical Skills; ANTSdk, Anesthesiologists’ Non-Technical Skills in Denmark; AOTP, Assessment of Obstetrical Team Performance; ASNTS, Anesthesiology students’ Non-Technical Skills; BARS, Behaviorally Anchored Rating Scale; CALM, Concise Assessment of Leader Management; GAOTP, Global Assessment of Obstetrical Team Performance; HFRS, Human Factors Rating Scale; MHPTS, Mayo High Performance Team Scale; NOTSS, Non-Technical Skills for Surgeons; NTS, Non-Technical skills; NTS-NAS, Non-Technical Skills-Nursing Assessment Scale; OSANTS, Objective Structured Assessment of Non-Technical Skills; OSCAR, Observational Skill-based Clinical Assessment tool for Resuscitation; Ottawa GRS, Global Rating Scale; STAT, Simulation Team Assessment Tool; T-NOTECHS, Non-technical skills scale for trauma; TEAM, Team Emergency Assessment Measure; TPOT, Team Performance Observational Tool.

**TABLE 2:** Methodological quality of the included studies by measurement properties of NTS instruments.

NTS instruments (references)		Population	Scenario	Instrument design	Content validity	Risk of bias							
	
						Structural validity	Internal consistency	Cross cultural validity	Reliability	Measurement error	Criterion validity	Construct validity	Responsiveness
**ANTS**	(42)	50 anesthesiologists	Anesthesiology	I	D		D		I	V			
	(65)	70 anesthesiology R	Operating room emergency	I	D		I		V			A	A
**ANTSdk**	(66)	31 HCPs	General surgery	I									
	(67)	19 anesthesiologists	Anesthesiology	I					V				A
**ASNTS**	(17)	21 anesthesiology R	Anesthesiology/ emergency	D	D				D				
**Assessment of EM physicians’ NTS**	(55)	148 HCPs	Emergency	D	A								
**AOTP-** **GAOTP**	(43)	72 obstetrical team members	Obstetric emergencies	I	D		I		V				
**BARS tool**	(41)	Anesthesiology R and NS	Pediatric emergency	I					V				A
**CALM**	(68)	40 HCPs	Pediatric emergency	D	D				D				
**GRS**	(12)	59 R	Emergency	I	D		D		V				
	(64)	16 N, 6 O, 6 anesthesiologists, and 6 R	Obstetric emergencies	I			I		V				
	(69)	28 R	Medical/Obstetric Emergency	I			I	I	V				
	(65)	70 anesthesiology R	Operating room emergency	I	D		I		V			A	A
**HFRS**	(64)	16 N, 6 O, 6 anesthesiologists, and 6 R	Obstetric emergencies	I			I		V				
**LOSA**	(70)	27 surgical R	Respiratory emergencies	D			I		I			V	
**MHPTS**	(71)	107 N-P	Medical/anesthesio-logy Emergency	I		A	A		A				A
**NOTSS**	(72)	27 consultant surgeons	General/orthopedic/ cardiac surgery	D									
	(73)	44 P	General/orthopedic surgery	I					V		V		
**NTS-NAS**	(13)	177 NS		D	A	I	A						
**OSANTS**	(40)	Surgical R	General surgery	I			I		V			V	I
**OSCAR**	(38)	Anesthesiologists, P-N	Emergency	I	A		V		V				
**STAT**	(39)	Pediatric R and experts	Pediatric septic shock	I	D				V			A	
**T-NOTECHS**	(37)	2 surgeons, 1 intensivist, and 2 N	Trauma	I			I		A				
	(74)	193 HCPs	Trauma	D		A	D	V	V				
**TEAM**	(44)	6 resuscitation experts	Emergency	D	D	D	V		V				
	(45)	35 HCPs	Emergency	D		A	V		V				
	(46)	151 HCPs	Obstetric emergencies	I		A	V		V				
**TPOT**	(57)	72 MS-NS	Trauma	I			V		V			V	

The table includes the objective and population of each article. Methodological quality of the included studies by measurement properties is evaluated as very good (V), adequate (A), doubtful (D), inadequate (I) by the COSMIN method. Empty cells indicate that that measurement property (or part of it) was not performed. ANTS, Anesthetists’ Non-Technical Skills; ANTSdk, Anesthesiologists’ Non-Technical Skills in Denmark; ASNTS, Anesthesiology students’ Non-Technical Skills; AOTP, Assessment of Obstetrical Team Performance; BARS, Behaviorally Anchored Rating Scale; CALM, Concise Assessment of Leader Management; GAOTP, Global Assessment of Obstetrical Team Performance; HCPs, Healthcare professionals; HFRS, Human Factors Rating Scale; MHPTS, Mayo High Performance Team Scale; MS, Medicine students; NOTSS, Non-Technical Skills for Surgeons; NS, Nursing students; NTS, Non-Technical skills; NTS-NAS, Non-Technical Skills-Nursing Assessment Scale; N, Nurses; O, Obstetricians; OSANTS, Objective Structured Assessment of Non-Technical Skills; OSCAR, Observational Skill-based Clinical Assessment tool for Resuscitation; Ottawa GRS, Global Rating Scale; P, Physicians; R, Residents; STAT, Simulation Team Assessment Tool; T-NOTECHS, Non-technical skills scale for trauma; TEAM, Eam, Emergency Assessment Measure; TPOT, Team Performance Observational Tool.

### Quality assessment

#### Measurement properties of assessment tools

The development of the NTS instruments were reported in 14 studies. The quality of the development of the NTS instruments was doubtful or inadequate, due to missing substantial elements for an adequate development process according to the COSMIN method. The 26 included studies reported NTS instrument measurement properties, primarily on: reliability (*n* = 22; 85%), internal consistency (*n* = 17; 65%), and content validity (*n* = 11; 42%). For a total of 19 NTS assessment instruments, the following measurement properties were reported: content validity (*n* = 19), internal consistency (*n* = 12), reliability (*n* = 17), construct validity and responsiveness (*n* = 6), structural validity (*n* = 4), cross cultural validity (*n* = 2), criterion validity (*n* = 1), and measurement error (*n* = 1). The methodological quality of the included studies by measurement properties of NTS instruments is reported in Table 2.

**FIGURE 2:**
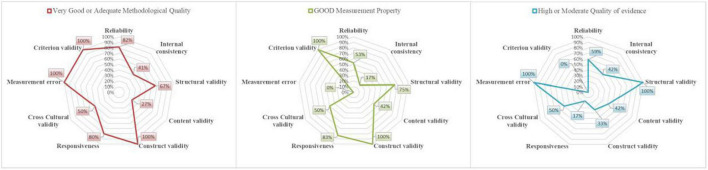
Quality assessment. The assessed measurement properties of 19 NTS instruments to which the plotted lines refer, are distributed around the radar. The red line in the first chart plots the proportion of studies with a very good or adequate methodological quality, over a total of 26 studies. The green line in the second chart represents the proportion of instruments with good measurement properties. The blue line in the third chart shows the proportion of instruments with high or moderate evidence quality. For example, content validity was of very good or adequate methodological quality in 27% of the included articles (3/11 studies), instruments with good measurement properties of content validity were 42% (8/19 instruments) and instruments with high or moderate evidence quality of content validity were 42% (8/19 instruments).

Overall ratings of the measurement properties of the NTS instruments and the quality of evidence are reported in Table 3. Figure 2 shows the overall methodological quality of the studies included in this review, the good measurement properties and the quality of evidence (≥ moderate) of the NTS instruments reported in this review. In four studies, the usability and feasibility of the NTS instruments Anesthetists’ Non-Technical Skills (ANTS), Anaesthesiology Students’ Non-Technical Skills (AS-NTS), Assessment of Obstetrical Team Performance (AOTP)/Global Assessment of Obstetrical Team Performance (GAOTP) and Team Emergency Assessment Measure (TEAM) was assessed through a survey administered to the users (17, 42–44).

**TABLE 3:** Evaluation of the measurement properties and quality grading of the evidence of NTS instruments.

NTS instruments	Content validity		Structural validity		Internal consistency		Cross cultural validity		Reliability		Measurement error		Criterion validity		Construct validity		Responsiveness	
									
	Overall rating	QoE	OR	QoE	OR	QoE	OR	QoE	OR	QoE	OR	QoE	OR	QoE	OR	QoE	OR	QoE
**ANTS**	+	M			?	L			–	M	?	M			+	L	+	L
**ANTSdk**	–	VL							+	M							+	L
**ASNTS**	+	M							+	VL								
**Assessment of EM physicians’ NTS**	+	H																
**BARS tool**	–	VL							+	M							+	L
**AOTP-GAOTP**	±	L			?	VL			+	L								
**CALM**	+	M							–	VL								
**GRS**	?	L			?	L	+	VL	±	M					+	L	+	L
**HFRS**	–	VL			?	VL			–	VL								
**LOSA**	–	VL			?	VL			?	VL					+	L		
**MHPTS**	–	VL	?	M	+	M			?	L							+	M
**NOTSS**	–	VL							+	L			+	L				
**NTS-NAS**	+	H	–	VL	?	M												
**OSANTS**	–	VL			?	VL			+	M					+	M	?	VL
**OSCAR**	+	H			?	M			+	M								
**STAT**	+	M							+	M					+	L		
**T-NOTECHS**	–	VL	+	M	?	L	?	M	±	M								
**TEAM**	+	M	+	H	+	H			±	M								
**TPOT**	–	VL			–	L			+	M					+	M		

The measurement properties of the NTS instruments are evaluated as sufficient (+), insufficient (–), inconsistent (±), or indeterminate (?) based on the coherent results obtained in one or more studies, by criteria for good measurement properties of the COSMIN method. The quality of the evidence is graded as high (H), moderate (M), low (L), very low (VL) evidence using a modified GRADE approach, based on the COSMIN method. Empty cells indicate that that measurement property (or part of it) was not performed. ANTS, Anesthetists’ Non-Technical Skills; ANTSdk, Anesthesiologists’ Non-Technical Skills in Denmark; AOTP, Assessment of Obstetrical Team Performance; ASNTS, Anesthesiology students’ Non-Technical Skills; BARS, Behaviorally Anchored Rating Scale; CALM, Concise Assessment of Leader Management; GAOTP, Global Assessment of Obstetrical Team Performance; HFRS, Human Factors Rating Scale; MHPTS, Mayo High Performance Team Scale; NOTSS, Non-Technical Skills for Surgeons; NTS, Non-Technical skills; NTS-NAS, Non-Technical Skills-Nursing Assessment Scale; OR, Overall rating; OSANTS, Objective Structured Assessment of Non-Technical Skills; OSCAR, Observational Skill-based Clinical Assessment tool for Resuscitation; Ottawa GRS, Global Rating Scale; QoE, Quality of evidence; STAT, Simulation Team Assessment Tool; T-NOTECHS, Non-technical skills scale for trauma; TEAM, Team Emergency Assessment Measure; TPOT, Team Performance Observational Tool.

#### Recommendations

Of the 19 instruments, according to the COSMIN criteria, the Team Emergency Assessment Measure (TEAM) can be recommended for the assessment of NTS as it has sufficient evidence of content validity and at least low quality evidence for sufficient internal consistency. TEAM was developed in Australia for trained observers to rate team performance during simulated resuscitations and deliver constructive debriefing sessions. The instrument has 3 domains (leadership, teamwork, and task management) and 11 items. TEAM was studied in the medical emergency and obstetric/gynecologic settings. TEAM’s content validity (content validity index = 0.96) and internal consistency was reported to be high (ranging from Cronbach’s α = 0.85–0.92) while its reliability, was moderate-to-high (ICC = 0.66–0.98) (44–46). Overall acceptability and satisfaction with the use of TEAM was high, including design and observability of the teamwork skills. In one study, the item “team morale” was rated as difficult to determine, particularly for raters without previous experience with resuscitation events (44).

All the other instruments included in this review require further research to assess their quality in order to be recommended for use during simulation training. Eight NTS instruments had a positive overall rating of their content validity with at least a moderate quality of evidence, but would need further testing to be recommended, regarding construct validity and internal consistency. None of the instruments included in this review were considered not recommendable for HFS training because there was no high-quality evidence that confirmed the inadequacy of their psychometric properties.

## Discussion

This systematic review applied the COSMIN methodology to assess the psychometric properties of instruments measuring NTS. A total of 19 instruments to evaluate NTS during high fidelity simulations were identified. One instrument, TEAM, fulfilled the psychometric testing requirements for the recommendation of its use during HFS training according to the COSMIN criteria. All the other instruments require further testing as they did not report sufficient evidence of content validity or at least show low-quality evidence for sufficient internal consistency. None of the instruments were considered not recommendable, suggesting that there is margin to further investigate and report the essential measuring properties required for recommending their use.

Patient safety is receiving increasing attention in HCPs’ curricula. The importance of NTS training is consistently emerging as an essential component of safety competence for HCPs (47–49). Measuring NTS during HFS training through validated instruments is essential to monitor their development and improve their awareness among medical, nursing students and HCPs (50). The effectiveness of HFS programs is based on their ability to achieve their educational goals, which may include both technical and NTS. While TS are commonly evaluated through standardized instruments, NTS are seldom evaluated (51–53). Increasing evidence of the effect of simulation programs on the acquisition and maintenance of NTS in HCPs is essential for resource allocation and planning simulation training, including simulation content, length and frequency, and target groups. Reliable and valid NTS measurement instruments can set the stage to accurately measure change of essential CRM behaviors during simulation training.

In this study, we found that the methodological quality of instrument development was doubtful or poor for all studies, and only 1 instrument (5%) could be recommended. This finding has two main implications. First, in the domain of the assessment of NTS, instrument development and content validity studies need to rigorously report the application of a consistent methodological approach according to accredited reporting guidelines to demonstrate process validity and reliability. For this review, we used the COSMIN method as it sets clear criteria for instrument development and psychometric testing of the instruments’ measuring properties using Delphi consensus based procedures. NTS assessment instruments developed before the COSMIN criteria were published (in 2010) referred to standards of prior psychometric evaluation tools. Those studies are more likely to be less compliant with the detailed and rigorous COSMIN criteria (54). Future research should aim at following recommended criteria for the evaluation of psychometric tools to safeguard the quality, validity and reliability of NTS measuring instruments for future use in the simulation setting.

Second, the research gap in this domain is wide, as there is limited evidence on essential aspects of validity and reliability for most instruments reported in this review, requiring further research. Content validity is the most important measurement property of a measuring instrument as it refers to item relevance, comprehensiveness and comprehensibility with respect to the construct of interest and study population (28). The eight instruments (13, 17, 38, 39, 42, 44, 55, 68) that reported a positive rating of content validity should be further evaluated at least for structural validity and internal consistency to determine a recommendation for their use.

Most NTS instruments were tested for reliability. Reliability of instrument domains and items resulted lower for concepts that might be more difficult to translate into an observable behavior, such as situational awareness, teamwork or team morale (45). NTS definition through a shared framework including situated examples of expected behaviors in different settings, as reported for some instruments (17, 38, 55–57) is essential to promote users’ shared understanding of the behaviors to observe and the accuracy of the application of NTS instruments.

NTS are comprehensive concepts characterized by their complexity, interconnectedness, evolving scope and meaning, irrespective of the scenario been simulated. The instruments included in this review reported primarily NTS domains described as “leadership” and “teamwork,” followed by “communication” and “situation awareness.” Only about half of the instruments used “decision making” and “task management,” which are often included in “leadership,” “teamwork” or “situation awareness.” Leadership is mostly regarded in relation to managing a team or organization (58) but can also be defined as a set of personal skills or traits, or focusing on the relation between leaders and followers (59, 60). Leaders in healthcare should have both the technical and social competences to exercise effective situational leadership and a flexible approach to patient management (61). Markers of effective teamwork include: calling for help early, establishing clear roles and leadership, employing team-oriented communication techniques, establishing a team situational awareness, effective decision making, and maintaining an adequate group climate (62). On the other hand, individual and team situation awareness is a complex dynamic process to maintain awareness of a critical clinical situation based on perception, comprehension and projection. A closer relationship with the environment, taking into account contextual factors, determines a distributed situational awareness. Finally, communication can be intended as a neutral means to share information between individuals or more as a means to structure social processes, including leadership and followership, and share mental models. Failures in communication have been found to be a leading cause of errors in healthcare determining the lack of sharing of a common mental model and understanding of the patient’s conditions. Closed-loop and direct communication is an essential goal for reliability in healthcare. Establishing clear mental models on NTS is a prerequisite for content validity of instruments that evaluate NTS.

Simulation instructors, while assessing NTS, face the need to strike a balance between capturing the nuances of non-technical behavior in managing simulated patient emergencies and synthesis. ANTS, AS-NTS, AOTP/GAOTP, and TEAM were reported to have a high usability rate. Efficiency, satisfaction and effectiveness are essential domains of instrument usability (63), which should be applied to instruments devised to assess NTS. Simplicity, ease of use, and accuracy are essential to reduce assessment errors and to increase NTS evaluation during simulation training. Moreover, the NTS instruments included in this review were developed and used for emergency scenarios in different clinical settings. Some instruments present examples of expected NTS behavior or are directed to specific teams, to increase usability and transferability in specific settings (38, 40, 43, 64).

### Limitations

This review has some limitations. We included instruments for the evaluation of NTS during simulation training with the exclusion of instruments devised for observing NTS in the clinical setting. While the simulation setting is where HCPs and healthcare students’ NTS training takes place, the clinical setting is where NTS ultimately should be practiced. In order to evaluate the effect of CRM simulation training on healthcare practices, NTS instruments should be validated both on simulation and real settings to be able to compare and evaluate the uptake of those skills.

## Conclusion

Out of a large variety of published instruments devised to assess NTS, the TEAM instrument can be recommended for use during HFS. Evidence is still limited on essential aspects of validity and reliability of all the other NTS instruments included in this review. Further research is needed to establish their performance in order to be reliably used for HFS.

## Data availability statement

The original contributions presented in this study are included in the article/Supplementary material, further inquiries can be directed to the corresponding author.

## Author contributions

OG conceived and designed the study. OG, KRT, CG, SE, VS, IE, and FC carried out the systematic literature review. OG, KRT, CG, SE, VS, AV, and FC involved in data analysis and performed the quality assessment. OG, KRT, CG, VS, and FC drafted the manuscript. All authors revised the manuscript and approved the final version as submitted.
