# Supplementary material for: Instruments to evaluate non-technical skills during high fidelity simulation: A systematic review
Source: Front Med (Lausanne). 2022 Nov 3;9:986296. doi: 10.3389/fmed.2022.986296 (PMC9669714; doi:10.3389/fmed.2022.986296)
Supplement: Supplementary file 1 [file Table_1.DOCX]

Supplementary Material

**Supplementary Table 1:** Population, Interest and Outcome Table with search strategy

| **Database** | **Categories** | **Keywords** | **Filters** |
| --- | --- | --- | --- |
| PubMed | **P** | (("Simulation Training"[Mesh]) OR ("High Fidelity Simulation Training"[Mesh])) AND | Human |
|  | **I** | ((human factor*) OR (resource management) OR (stress management) OR (resource utilization) OR (task management) OR (human error) OR (non-technical skill*) OR (nontechnical skill*) OR ("Intersectoral Collaboration"[Mesh]) OR ("Communication"[Mesh]) OR ("Crew Resource Management, Healthcare"[Mesh]) OR ("Leadership"[Mesh]) OR ("Decision Making"[Mesh]) OR (team work) OR (team-work) OR (teamwork) OR (situation awareness)) AND |  |
|  | **O** | ((assessment) OR (evaluation)) |  |
| Cinahl | **P** | ((Simulation Training OR High Fidelity Simulation Training)) AND | Human |
|  | **I** | ((human factor*) OR (resource management) OR (stress management) OR (resource utilization) OR (task management) OR (human error) OR (non-technical skill*) OR (nontechnical skill*) OR (Intersectoral Collaboration) OR (Communication) OR (Crew Resource Management) OR (Leadership) OR (Decision Making) OR (team work) OR (team-work) OR (teamwork) OR (situation awareness)) AND |  |
|  | **O** | ((assessment) OR (evaluation)) |  |
| Cochrane | **P** | ((Simulation Training OR High Fidelity Simulation Training)) AND | Human |
|  | **I** | ((human factor*) OR (resource management) OR (stress management) OR (resource utilization) OR (task management) OR (human error) OR (non-technical skill*) OR (nontechnical skill*) OR (Intersectoral Collaboration) OR (Communication) OR (Crew Resource Management) OR (Leadership) OR (Decision Making) OR (team work) OR (team-work) OR (teamwork) OR (situation awareness)) AND |  |
|  | **O** | ((assessment) OR (evaluation)) |  |

**Supplementary Table 2**: NTS’ instruments and included studies ordered by type of scenario

| **NTS instrument** | **Author, year and country** | **Objectives** | **Scenario** |
| --- | --- | --- | --- |
| **ANTS** | Fletcher, G., et al. (2003) (UK) | To examine validity, reliability and usability | Anaesthesiology |
| **ANTSdk** | Jepsen, R., et al. (2016) (Denmark) | To collect validity evidence for response process, reliability and internal structure | Anaesthesiology |
| **ASNTS** | Moll-Khosrawi, P., et al. (2019) (Germany) | To develop and validate the AS-NTS | Anaesthesiology/  Emergency |
| **GRS** | Kim, J., et al. (2006) (Canada) | To develop and validate the Ottawa GRS | Emergency |
| **Assessment of EM physicians’ NTS** | Flowerdew, L, et al. (2012) (UK) | To develop and content validate a behavioural marker system | Emergency |
| **OSCAR** | Walker, S., et al. (2011) (UK) | To develop and ensure validity, reliability, and feasibility | Emergency |
| **TEAM** | Cooper, S., et al. (2010) (Australia) | To develop a valid, reliable and feasible teamwork assessment measure | Emergency |
| **TEAM** | Freytag, J., et al. (2019) (Germany) | To compare novice and expert ratings using TEAM | Emergency |
| **OSANTS** | Dedy, N.J., et al. (2015) (Canada) | To develop and validate an evidence-based  and reliable tool | General surgery |
| **ANTSdk** | Jepsen, R., et al. (2015) (Denmark) | To customise the ANTS for Danish anaesthesiologists | General surgery |
| **NOTSS** | Yule, S., et al. (2006) (UK) | To identify surgeons ’ NTS and to develop a skills taxonomy and behavioural rating system | General/Orthopaedic/  Cardiac surgery |
| **NOTSS** | Yule, S., et al. (2008) (UK) | To evaluate the reliability | General/Orthopaedic surgery |
| **MHPTS** | Malec, J.F., et al. (2007) (USA) | To examine the psychometric characteristics | Medical/Anaesthesiology Emergency |
| **GRS** | Franc, J.M., et al. (2017) (Italy) | Evaluation of the reliability of the Italian version of the GRS | Medical/Obstetric Emergency |
| **AOTP-GAOTP** | Tregunno, D., et al. (2009) (Canada) | To develop two midwifery rating scales: AOTP and the GAOTP | Obstetric emergencies |
| **GRS** | Morgan,P.J., et al. (2007) (Canada) | To determine whether an adaptation of the HFRS and GRS could be used to reliably assess obstetric team performance | Obstetric emergencies |
| **HFRS** | Morgan,P.J., et al. (2007) (Canada) | To determine whether an adaptation of the HFRS and GRS could be used to reliably assess obstetric team performance | Obstetric emergencies |
| **TEAM** | Carpini, J.A., et al. (2021) (Australia) | To evaluate the psychometric properties | Obstetric emergencies |
| **ANTS** | Jirativanont, T., et al. (2017) (Thailand) | To evaluate the validity of the ANTS and Ottawa GRS | Operating room emergency |
| **GRS** | Jirativanont, T., et al. (2017) (Thailand) | To evaluate the validity of the ANTS and Ottawa GRS | Operating room emergency |
| **BARS tool** | Watkins, S., (2017) (USA) | To estimate the reliability and compare with ANTS | Paediatric emergency |
| **CALM** | Nadkarni L.D., et al. (2018) (USA) | To develop and validate a formative feedback instrument | Paediatric emergency |
| **STAT** | Reid, J., et al. (2012) (USA) | To develop and evaluate the inter-rater reliability and validity | Paediatric septic shock |
| **LOSA** | Moorthy, K., et al. (2005) (UK) | To evaluate the interrater reliability | Respiratory emergencies |
| **T-NOTECHS** | Steinemann, S., et al. (2012) (USA) | To evaluate the reliability and correlation of T-NOTECHS with clinical performance parameters | Trauma |
| **T-NOTECHS** | Repo, J.P., et al. (2019) (Finland) | To evaluate the translatability  into Finnish,  and to investigate the psychometric properties | Trauma |
| **TPOT** | Zhang, C., et al. (2015) (USA) | To decrease the subjectivity of TPOT and determine the psychometric properties | Trauma |
| **NTS-NAS** | Pires, S.M.P., et al. (2018) (Portugal) | To construct, develop and validate |  |
